# Diet in treatment of autism spectrum disorders

**DOI:** 10.3389/fnins.2022.1031016

**Published:** 2023-07-10

**Authors:** Sabiha Alam, Cara J. Westmark, Elizabeth A. McCullagh

**Affiliations:** ^1^Department of Integrative Biology, Oklahoma State University, Stillwater, OK, United States; ^2^Department of Neurology, University of Wisconsin, Madison, WI, United States; ^3^Molecular and Environmental Toxicology Center, University of Wisconsin, Madison, WI, United States

**Keywords:** autism spectrum disorders, diet, intervention, Fragile X Syndrome, probiotic, prebiotic, ketogenic

## Abstract

Altering the diet to treat disease dates to c. 400 BC when starvation was used to reduce seizures in persons with epilepsy. The current diversity of symptomology and mechanisms underlying autism spectrum disorders (ASDs) and a corresponding lack of disorder-specific effective treatments prompts an evaluation of diet as a therapeutic approach to improve symptoms of ASDs. In this review article, we summarize the main findings of nutritional studies in ASDs, with an emphasis on the most common monogenic cause of autism, Fragile X Syndrome (FXS), and the most studied dietary intervention, the ketogenic diet as well as other dietary interventions. We also discuss the gut microbiota in relation to pre- and probiotic therapies and provide insight into future directions that could aid in understanding the mechanism(s) underlying dietary efficacy.

## Introduction

Fragile X Syndrome (FXS) is a neurodevelopmental disorder characterized by intellectual disability, autistic-like behaviors and seizures (Hagerman and Hagerman, 2002). Children exhibit delayed sitting, walking or talking as well as social and behavioral impairments, i.e., not making eye contact, anxiety, aggression, hand flapping and attention deficits. Characteristic physical features include large ears, sunken chest, and enlarged head and testicles (McLennan et al., 2011; Marlborough et al., 2021). FXS is an inherited disorder that results from a CGG-trinucleotide repeat expansion mutation in the fragile X messenger ribonucleoprotein 1 (*FMR1*) gene located at Xq27:3 on the X-chromosome. An expansion greater than 200-CGG repeats leads to inactivation of the *FMR1* gene and loss of its protein product fragile X messenger ribonucleoprotein (FMRP) (Loesch et al., 2004; Hagerman et al., 2011; Saldarriaga et al., 2014; Ciaccio et al., 2017; Mor-Shaked and Eiges, 2018). Lack of FMRP results in FXS-related cognitive deficits and behavioral issues (Garber et al., 2008).

FXS is the most prevalent genetic cause of autism spectrum disorders (ASDs) (Budimirovic and Kaufmann, 2011). The etiology of ASDs is genetic and multifactorial caused by mutations in as many as 1,000 genes and influenced by environmental factors (Bagni and Zukin, 2019). The environmental and other risk factors contributing to ASDs include parental age, maternal nutritional and metabolic status, infection during pregnancy, prenatal stress, and exposure to certain toxins, heavy metals, or drugs. Specifically, increased maternal age, nutrient deficiencies, and trace element imbalance also affect proper brain development sometimes resulting in non-genetic ASDs (Hagmeyer et al., 2015; Karimi et al., 2017; Gialloreti et al., 2019).

FXS and other ASDs are neurodevelopmental disorders with overlapping symptoms providing strong phenotypes to test and identify treatments to mitigate core symptoms, i.e., clinical features including anxiety, aggression, anti-sociability, gut microbiota dysbiosis, and neuroinflammation (Lozano et al., 2016; Zheng et al., 2021). The gut microbiota affects social behaviors including feeding in animals leading to changes in overall nutrition (Pasquaretta et al., 2018). Existing literature suggests that the *Fmr1* gene (the homologous gene in mouse) can affect gut microbiota (Goo et al., 2020; Altimiras et al., 2021), although no clinical studies have been conducted to understand the link between *FMR1* gene expression and the human gut microbiome. Similarly, many experiments have been performed with the goal to understand the mechanisms of gut-brain pathologies in ASDs but there is no consensus on an overarching framework. This review aims to summarize main findings in nutrition and ASDs, with an emphasis on FXS, as well as provide insight into future directions that would aid in understanding complementary or differing mechanisms between these disorders and effects of interventions.

## Autism spectrum disorders and gut microbiota

Over the past few decades, the gut microbiota has gained tremendous attention. Indeed, gut microbiota affect many metabolic and neurobehavioral traits. Nutritional status depends on dietary choices and imbalanced diets can adversely modulate intestinal microbial diversity (Dinan et al., 2015). Microbial dysbiosis leading to gut microbial imbalance and gastrointestinal (GI) disturbances are strongly associated with ASDs, including FXS. When diversity of the GI microbiome is disrupted, it can play a critical role in advancing metabolic dysfunction such as type 2 diabetes, cancer, hypertension, and inflammatory bowel diseases (Manor et al., 2020), as well as alter nutrient digestion and absorption, ultimately leading to further changes in feeding and social behaviors (Pasquaretta et al., 2018). Healthy gut bacteria produce digestive enzymes which are beneficial in breaking down complex carbohydrates and proteins and thus increase nutrient digestion and absorption. Altered gut microbiota influence certain aberrant behaviors in ASDs by modulating gut-brain signaling through changing intestinal motility, sensitivity, and mucosal immunity (Matricon et al., 2012). Therefore, in-depth research is necessary to understand the mechanisms, followed by the establishment of therapeutic avenues to restore microbial balance in ASDs. Since nutrition is a crucial factor in gut-brain health, it is an important variable to consider when testing new interventions to treat core social, behavioral, and physiological traits of ASDs.

ASD phenotypes have been shown to be mitigated in response to dietary supplementation or behavioral interventions. In this review article, we discuss nutritional interventions that have been studied in human and animal models of ASDs, including FXS, and their effects on mitigating core symptoms.

## Dietary approaches in autism spectrum disorders

Nutrition plays a pivotal role in the body’s growth and maintenance. Previous studies have shown that nutrition can influence the core symptoms of ASDs including FXS: however, the nutritional status of individuals affected by ASDs remains to be precisely assessed. Imbalanced dietary intake may cause nutrition deficiencies or even toxicity, particularly in the context of genetic mutations that have not been studied in relation to metabolic defects. Therefore, we need to focus on two approaches:

(1). Supplementation of macro or micronutrients that are deficient in ASDs, and (2). Avoiding nutrients and bioactive food components that exacerbate adverse phenotypes or cause illness. Among the micronutrients, different vitamins and minerals including fat-soluble vitamins (vitamin A, vitamin D), water-soluble vitamins (vitamin C, folic acid, vitamin B6, vitamin B12), and minerals (copper, zinc) are important for overall nutrition. In addition, dietary fatty acids, prebiotics, and probiotics have been used as supplements to treat ASDs (Kawicka and Regulska-Ilow, 2013). In contrast, some foods (casein or gluten) cause intolerance and contribute to worsening of ASD symptoms and can be avoided or excluded from the diet (Table 1).

**TABLE 1:** Nutritional interventions in ASDs.

Nutritional interventions	Study type	Results	References
Alpha-tocopherol	Preclinical	Reduced oxidative stress, behavioral abnormalities, learning deficits in FXS mice	de Diego-Otero et al., 2009
Vitamin B6+ magnesium (Mg)	Clinical	Improved core symptoms of ASD	Nye and Brice, 2005
Vitamin B12	Clinical	Reduced ASD related metabolic dysfunctions, irritability, aggression in children with ASD	Malhotra et al., 2013
Vitamin D and Omega-3 fatty acids	Clinical	Reduced hyperactivity and irritability among autistic children	Mazahery et al., 2019
Folinic acid	Clinical	Improved social behavior and cognition deficits in children with ASD	Frye et al., 2013
Lithium	Preclinical	Mood stabilizers in ASD; reduced hyperactivity, anti-social behaviors, cognition deficits in FXS mice	Liu et al., 2011; Mintz and Hollenberg, 2019
Omega-3 fatty acids	Preclinical	Improved ultrasonic vocalizations and social discriminations in rat models with ASD and FXS; improved emotion, non-spatial memory, social interactions and neuroinflammation in FXS mice	Pietropaolo et al., 2014; Schiavi et al., 2022
Breast milk	Retrospective clinical	Reduced incidence of autism in FXS	Westmark, 2021b
Specific carbohydrate diet	Clinical	Improved GI symptoms, behavioral abnormalities, and nutritional status in a child with both FXS and ASD	Barnhill et al., 2020
Ketogenic diet	Preclinical	Improved epilepsy, repetitive behavior, social skills, impaired learning skill in mice with ASD; Improved social exploration and interactions in rodents; reduced hyperactivity and seizures in mouse model of FXS	Ruskin et al., 2013; Castro et al., 2017; Westmark et al., 2020b
Soy vs. casein-based rodent diet	Preclinical	Increased seizures and weight gain with soy	Westmark et al., 2013, Westmark et al., 2022
Soy vs. casein-based infant formulas	Retrospective clinical	Increased seizures, autism, GI problems and allergies in ASD and FXS populations with soy	Westmark, 2013, 2014a, 2017, 2021a; Westmark et al., 2020a

## Micronutrients and autism spectrum disorders

Recent studies indicate significant advances in the early detection of ASDs and preclinical research and clinical trials have begun to assess the efficacy of non-pharmacological therapies at earlier ages. Micronutrients may be an important component of ASD etiology, though many results are conflicting. For example, in a 2022 systematic review, authors describe that vitamin D can improve hyperactivity in ASD, but their research did not show enough beneficial evidence for improving ASD-related symptoms (Li et al., 2022). In addition, vitamin D with omega-3 supplementation showed beneficial effects in treating hyperactivity and irritability in children with ASD (Mazahery et al., 2019).

Alpha-tocopherol, known as vitamin E, is a free radical scavenger with potential in treating FXS. *Fmr1* knockout (KO) mice were supplemented with alpha-tocopherol, which resulted in a reduction of free radicals causing less oxidative stress along with cytokine production and reduced macroorchidism. The alpha-tocopherol treatment also improved behavioral abnormalities and reduced learning deficits in *Fmr1*^KO^** mice (de Diego-Otero et al., 2009). Folinic acid deficiency leads to impairment in cellular methylation increasing oxidative stress in ASDs (Frye et al., 2013). In a recent study, children treated with folic acid supplementation, and involved in structured teaching, showed significant improvements in social, behavioral, and cognitive deficits (Sun et al., 2016). Maternal dietary intake of folic acid may also reduce the risk of developing ASD in offspring (Liu et al., 2022). Additionally, vitamin B12, also known as cobalamin, was shown to be effective in treating ASD-related metabolic dysfunction and improved clinical symptoms like irritability, aggression, and other aberrant behaviors in children affected by ASD (Malhotra et al., 2013). Vitamin B6 and magnesium have a long history of treating core symptoms of ASDs, but the small number of studies, differences in methodological study design, selection of study subjects, and small sample sizes have prevented their implementation for treatment (Nye and Brice, 2005). Lithium is a micronutrient provided mostly through the diet from fruits, and vegetables. Lithium has been used as a mood stabilizer in adolescents and adults with ASD (Mintz and Hollenberg, 2019). Additionally, it is effective in treating FXS phenotypes, including hyperactivity, anxiety, anti-socialism, and cognitive deficits, in *Fmr1*^KO^** mice (Liu et al., 2011). A personalized medicine approach will be required to implement micronutrient-mediated therapy for ASDs, particularly considering the contradictory evidence regarding the efficacy of population level folic acid supplementation and neural tube defects (Murphy and Westmark, 2020).

## Omega-3 fatty acids and autism spectrum disorders

Omega-3 (Ω-3) fatty acids (FA) are essential for normal growth and maintenance of body functions; therefore, many studies have been performed to test their efficacy in mitigation of ASD traits. Ω-3 fatty acids were administered to *Fmr1*^KO^** mice and rats and rescued ultrasonic vocalizations, social discrimination, and hyperactivity (Nolan et al., 2020, 2022; Schiavi et al., 2022). FXS mice treated with Ω-3 FAs also showed amelioration of alterations of emotion, social interaction, and non-spatial memory and rescue of inflammatory biomarkers (i.e., increased CD11b and CD45 in hippocampal CA1 and dentate gyrus, decreased IL-1β in CA3, increased IL-1β in prefrontal cortex, and increased TNF-α in CA1) (Pietropaolo et al., 2014). Considering that loss of FMRP is associated with increased utilization of lipids as an energy source (Leboucher et al., 2019), it is important to study the effects of specific FA on ASD phenotypes.

## Ketogenic diet and autism spectrum disorders

The classic ketogenic diet was introduced in 1921 to replace starvation in the treatment of epilepsy (Greener, 2014). The ketogenic diet is high in fat with moderate levels of protein and low carbohydrate. It mimics an Inuit diet where there are periods of low food availability and the body is forced to burn fat for energy, “ketosis.” Glucose is normally the sole energy source for the human brain, but ketones are produced and used for energy in ketosis. In addition to intractable epilepsy, ketone- rather than glucose-based metabolism may benefit other conditions. For example, the ketogenic diet is under study for the treatment of a wide range of disorders and conditions including Alzheimer’s disease, amyotrophic lateral sclerosis (ALS), anxiety, attention-deficit hyperactivity disorder (ADHD), ASDs, bipolar disorder, cancer, depression, diabetes, obesity, pain, Parkinson’s disease, schizophrenia, stroke, and traumatic brain injury (Evangeliou et al., 2003; Tai et al., 2008; Masino et al., 2009; Balietti et al., 2010; Frye et al., 2011; Jóźwiak et al., 2011; Stafstrom and Rho, 2012; Herbert and Buckley, 2013; Spilioti et al., 2013; Napoli et al., 2014; Garcia-Penas, 2016; Bostock et al., 2017; Cheng et al., 2017; Verrotti et al., 2017). There is growing interest in employing the KD for the treatment of ASDs, which are highly comorbid with epilepsy such that it has been proposed that epilepsy drives the development of ASD (Amiet et al., 2008; Hartley-McAndrew and Weinstock, 2010; Hagerman, 2013; van Eeghen et al., 2013). Thus, treatments that reduce seizure incidence have the potential to prevent the development of ASDs or decrease the severity of symptoms.

Recent studies in ASD rodent models indicate that the KD improves core behavioral symptoms albeit there were some sex and genotype-specific differences (Smith et al., 1991, 2016; Mantis et al., 2009; Ruskin et al., 2013, 2017a,b; Ahn et al., 2014; Verpeut et al., 2016; Castro et al., 2017; Dai et al., 2017; Kasprowska-Liśkiewicz et al., 2017). Preliminary studies in humans also indicate improvement in autistic behaviors in response to the KD, but sample sizes have been small (Evangeliou et al., 2003; Frye et al., 2011; Herbert and Buckley, 2013; Spilioti et al., 2013; Bostock et al., 2017). Despite these successes, the mechanism underlying success of the KD and ketosis is not understood, but most likely involves restoration of aberrant energy metabolism. Possible effectors include adenosine, ketones, lactate dehydrogenase, medium-chain fatty acids (MCFA), neurotrophic factors, O-linked-β-N-acetyl glucosamine (O-GlnNAc), and polyunsaturated fatty acids (PUFA); and affected processes include epigenetic and gene expression mechanisms, the GABAergic and cholinergic systems, inflammatory pathways, mitochondrial dynamics, oxidative stress, synaptic transmission, and the gut microbiome (Kossoff et al., 2009; Masino et al., 2009; Wallace et al., 2010; Freche et al., 2012; Stafstrom and Rho, 2012; Napoli et al., 2014; Newell et al., 2016a,b, 2017; Boison, 2017; Cheng et al., 2017; Mychasiuk and Rho, 2017; Augustin et al., 2018; Ahn et al., 2020). Overall, the consensus is that the animal studies are promising, the mechanism of action is not understood, and the evidence in humans is insufficient to form an opinion as to the efficacy or lack thereof of the KD intervention for the treatment of brain disorders including ASD.

Others have reviewed current nutritional approaches in managing ASDs, the fundamental metabolic processes that promote brain health, and case and clinical studies testing the KD in ASD (Boison, 2017; Bostock et al., 2017; Cekici and Sanlier, 2019; Li et al., 2021; Varesio et al., 2021; Yu et al., 2022). Here, we highlight a few case studies successfully employing the KD in the treatment of ASD and thus increasing the available evidence regarding the safety and efficacy of this treatment for ASD. In their 2017 study published in Metabolic Brain Disorders and entitled, *Ketogenic diet vs. gluten free casein free diet in autistic children: a case-control study*, El-Rashidy et al. (2017) conduct a case-control study in 45 children aged 3–8 years and diagnosed with ASDs based on Diagnostic and Statistical Manual of Mental Disorders, 5th Edition (DSM-5) criteria. The children were equally divided into 3 diet cohorts: the Modified Atkins diet (MAD), a gluten free casein free (GFCF) diet, and a balanced nutrition control group. They were evaluated at baseline and 6 months after introduction of the dietary intervention by the Childhood Autism Rating Scale (CARS) and the Autism Treatment Evaluation Test (ATEC). Five patients dropped out of the MAD cohort due to poor compliance with the diet. Both the MAD and GFCF diet groups exhibited significant improvement in CARS and ATEC scores in comparison to the control group albeit the MAD cohort scored better in cognition and sociability than the GFCF cohort. The GFCF cohort exhibited improvement in total CARS and ATEC scores of speeches and behavior. Sociability and cognition did not significantly improve in the GFCF cohort as assessed by ATEC.

In their paper published in Metabolic Brain Disease in 2018 and entitled, *Therapeutic use of carbohydrate-restricted diets in an autistic child; a case report of clinical and 18FDG PET findings*, Żarnowska et al. (2018) present a case study of a 6-year-old patient with high-functioning autism and subclinical epileptic discharges. The patient was diagnosed with early childhood autism, mental retardation, and ADHD using the Diagnostic and Statistical Manual of Mental Disorders, 4th Edition, Text Revision (DSM-IV-TR) criteria. A sleep-phase electroencephalogram (EEG) revealed bilateral, synchronous, and asynchronous centro-temporal spikes and spike-wave complexes, but behavioral epileptic-like events were not observed. The patient responded poorly to several behavioral and psychopharmacological treatments and was placed on the KD after significant brain glucose hypometabolism was observed by positron emission tomography (PET) with 18 fluoro-deoxyglucose (18FDG PET), which measures brain glucose metabolic rate. Baseline adapted CARS and Wechsler Intelligence Scale for Children-revised (WISC-R) scores indicated severe ASD, borderline intellectual disability, below average performance, and average verbal skills, respectively. A KD with a 2:1 ratio of fats to proteins plus carbohydrates was introduced and achieved optimal ketosis. After 1 month, the KD was switched to MAD, which is less restrictive than KD with no limits on calories or protein and the lower overall ketogenic ratio does not need to be maintained for all meals. MAD was maintained for 5 months with moderate levels of ketones before the patient was placed on a low glycemic index treatment (LGIT). Ketones were still detectable with the LGIT. Within 1 month of KD treatment, numerous behaviors (hyperactivity, attention span, abnormal reactions to visual and auditory stimuli, usage of objects, adaptability to changes, communication skills, fear, anxiety, and emotional reactions) as well as intellect improved and continued throughout the 16-month observation period. Evaluation at 16 months post-initiation of the KD indicated that improvement in the CARS with minimal-to-no symptoms of autism; the WISC-R Full Scale IQ and the Verbal Scale IQ improved to average ratings; and Performance Scale IQ improved to slightly lower than average.

In their paper entitled, *A modified ketogenic gluten-free diet with MCT improves behavior in children with autism spectrum disorder*, published in Physiology and Behavior in 2018, Lee et al. (2018) study a modified ketogenic gluten-free diet with supplemental medium chain triglycerides (KD/GF/MCT) in children ages 2–17 years. Subjects exhibited high to moderate level ASD symptoms as ascertained by the Autism Diagnostic Observation Schedule, 2nd edition (ADOS-2) and the Childhood Autism Rating Scale-Second Edition (CARS-2). Fifteen subjects completed the 3-month intervention period. There was significant improvement in core autism features as evidenced by the ADOS-2 Overall Total score and the Social Affect score, but the Restricted and Repetitive Behavior score was not significantly different. The CARS-2 score was significantly decreased.

In their paper entitled, *Metabolic framework for the improvement of* ASDs *by a modified ketogenic diet: a pilot study*, published in Journal of Proteome Research in 2020, Mu et al. (2020) classified subjects as high or low responders to ketogenic diet treatment based on reduction of the overall ADOS-2 score after 3 months treatment and found that high responders had higher levels of 3-hydroxybutyrate and ornithine, and lower levels of galactose compared to low responders. These data provide important insights regarding dietary responsive blood-based biomarkers in ASDs.

It remains to be determined if the positive effects associated with the KD in humans can be maintained after discontinuation of the diet. In rats, after 1 week cessation of the KD, social activity deficits returned to control levels (Kasprowska-Liśkiewicz et al., 2017), but in CD-1 mice, gestational exposure to ketogenic diet resulted in increased sociability and reduced depression in adult animals (Arqoub et al., 2020). It is important to note that there can be adverse side effects associated continuous maintenance on the KD. Children receiving the KD long-term are at higher risk for growth retardation, GI problems, carnitine deficiency, kidney stones, elevated lipids, cardiac abnormalities due to selenium deficiency, Fanconi renal tubular acidosis, pancreatitis, bone fractures, and micronutrient deficiencies (Kossoff et al., 2009). On a positive note, ketogenic diet was not associated with worse memory or hippocampal-dependent learning in mice (Ródenas-González et al., 2022), and rescued social deficits in a Shank3 mouse model of autism (Qin et al., 2021).

In summary, ketogenic diets have shown success in treating core symptoms i.e., epilepsy, repetitive behavior, intellectual impairment, language dysfunction, and social skills of ASDs. Children with ASDs treated with either a ketogenic diet or modified ketogenic diet showed improvements in social exploration and interaction, and repetitive behaviors, like rodent models of ASDs (Ruskin et al., 2013; Spilioti et al., 2013; Castro et al., 2017; El-Rashidy et al., 2017; Lee et al., 2018; Żarnowska et al., 2018). A recent study demonstrated that treating *Fmr1^KO^* mice with a ketogenic diet attenuated seizures and altered diurnal activity levels with sex and age specific effects (Westmark et al., 2020b). Overall, there is a growing body of knowledge from rodent and human studies demonstrating rescue of ASD phenotypes in response to the KD. The KD is a very restrictive diet that is difficult to maintain long-term; thus, elucidation of the mechanism underlying success of the diet may identify a less stringent therapeutic option without adverse side effects.

## Specific carbohydrate diet

Specific carbohydrate diets (SCD) have been used for the treatment of Crohn’s disease, celiac disease, ulcerative colitis, diverticulitis, and chronic diarrhea and are currently receiving attention for their potential in treating neurological disorders (Gottschall, 2004; Suskind et al., 2014; Obih et al., 2016). The purpose of the SCD protocol is to strictly avoid and eliminate all grains, lactose, and sucrose in the diet to restore and maintain a healthy gut microbial population (Gottschall, 1994). In a case study, SCD was introduced in a 4-year-old boy with both ASD and FXS-related symptoms. The SCD improved GI symptoms, behavioral phenotypes, and overall nutritional status (Barnhill et al., 2020).

## Dietary proteins and autism

The effects of soy-based diets have also been assessed on core phenotypes of ASDs. Maintaining *Fmr1^KO^* mice on single-source soy protein-based diets increased seizures and weight gain (Westmark et al., 2013, 2022). These preclinical rodent data elicit the hypotheses that infant feeding with soy protein-based formulas could be contributing to an increased prevalence of epilepsy and obesity in ASDs. Indeed, retrospective parental-reported use of soy-based infant formula is associated with increased prevalence of febrile seizures, simple partial seizures, epilepsy, allergies, asthma and ADHD as well as more severe deficits in language, communication, social overtures and hypersensitivity to environmental stimuli in the Simons Foundation Autism Research Initiative (SFARI) autism population (Westmark, 2013, 2014a,^2017^). In subjects with FXS, consumption of soy-based infant formula is associated with increased prevalence of autism, GI problems, allergies, and more severe autistic behaviors related to language and self-injurious behavior in the Fragile X Online Registry with Accessible Database (FORWARD) population (Westmark et al., 2020a; Westmark, 2021a). It should be noted that GI problems were the most frequent reason cited for switching to soy-based infant formula with a 25% reported usage rate in the FORWARD study population, which is significantly higher than the general population. In a national Korean population cohort, soy-based infant formula was associated with an increased prevalence of epilepsy and ADHD (Westmark, 2022). Possible mechanisms underlying soy-induced effects in ASDs could include an altered gut microbiome, metabotropic glutamate receptor 5 (mGluR_5_) / estrogen receptor dependent signaling, and/or activation of the immune system by soy bioactive components including phytoestrogens and agrochemicals (Westmark, 2014b). In terms of early life nutrition, breastfeeding is considered optimal and associated with numerous health benefits; however, the effects of breastfeeding, breast milk, and breast milk proteins on neurodevelopment are still understudied. Infants with FXS who were fed breast milk exhibited a reduced prevalence of autism with boys also having decreased GI problems and allergies (Westmark, 2021b). However, in those with GI problems or allergies, these comorbidities commenced significantly earlier than those not fed breast milk. Additional studies indicate that late weaning and exclusive breast milk protect against GI symptoms in infants at high risk for autism (Penn et al., 2016), and ASD prevalence prior to 36 months of age was lower in breastfed babies (Shamsedine et al., 2020).

## Prebiotic approaches in autism spectrum disorders

A prebiotic is a dietary fiber or complex sugar, which is degraded by gut microbiota resulting in short-chain FA circulating in the blood. Prebiotics can improve host health by stimulating the growth of beneficial gut microbiota (Davani-Davari et al., 2019).

The major prebiotic groups are fructooligosaccharides and galactooligosaccharides (Gibson and Roberfroid, 1995). A recent pilot clinical study performed on 2–11-year-old children with ASDs and GI-related pathologies, showed that probiotic supplementation (*Bifidobacterium infantis*) along with a prebiotic agent, Bovine Colostrum Product (BCP), improved overall aberrant behaviors and GI disturbances while decreasing inflammatory cytokines including IL-13 and TNF-alpha (Sanctuary et al., 2019). In another clinical study, the role of the prebiotic Bimuno^®^ galactooligosaccharide was evaluated in 30 children with ASDs while on an exclusion diet (casein and gluten). The treated group exhibited significant improvements in social behaviors and experienced less abdominal pain in bowel movements indicating the positive influence of prebiotic treatment in ASDs (Grimaldi et al., 2018; Table 2).

**TABLE 2:** Prebiotic interventions in ASDs.

Prebiotics	Study subject type	Results	References
Bovine Colostrum supplementation with probiotic (*Bifidobacterium infantis*)	Human	Reduced inflammation and GI disturbances with the improvement of aberrant behaviors related to ASD	Sanctuary et al., 2019
Bimuno^®^ galactooligosaccharide	Human	Improved social behavior and bowel movements	Grimaldi et al., 2018
Prebiotics (bee pollen and propolis)	Animal	Improved gut-microbial composition and neuroinflammation	Aabed et al., 2019
10% Oligofructose-enriched inulin	Animal	Worsened social behaviors in ASD model mice	Nettleton et al., 2021

In terms of preclinical studies, prebiotics (bee pollen and propolis) were assessed on neuroinflammation and gut dysbiosis in an autism rodent model [proprionic acid (PPA) treated golden Syrian hamsters]. This study showed significant improvements in gut-microbial composition and neuroinflammation in PPA-treated hamsters (Aabed et al., 2019). Interestingly, prebiotics (10% oligofructose-enriched inulin) worsened sociability in the BTBR (Black and Tan BRachyury) mouse model (Nettleton et al., 2021). In contrast, human studies showed increased social behavior and improved gut microbial composition and metabolism with prebiotic [Bimuno^®^ galactooligosaccharide (B-GOS^®^)] treatment in young children with ASDs on an exclusion (gluten and casein) diet (Grimaldi et al., 2018).

## Microbial approaches in autism spectrum disorders

Previous work demonstrated microbial-neural interactions as important for microbiota-based therapeutic interventions in neurodevelopmental disorders (Borre et al., 2014). Probiotics have gained support to restore gut microbial composition in hosts specifically for amelioration of gut inflammation (Hemarajata and Versalovic, 2013). Probiotics are living micro-organisms that elicit health benefits to the host when delivered in therapeutic doses (Abdellatif et al., 2020). The most common microorganisms used for probiotics are lactic acid bacteria and bifidobacteria along with non-pathogenic bacteria like *Streptococcus*, *Lactococcus*, and *Saccharomyces* (Plaza-Díaz et al., 2019). Further supporting the importance for microbiota in ASDs, clinical, preclinical, and experimental studies have shown alterations in gut microbial composition in ASDs in response to probiotics suggesting that this intervention may be an effective treatment (Garcia-Gutierrez et al., 2020; Table 3).

**TABLE 3:** Probiotic interventions in ASDs.

Probiotic strains	Study subjects	Results	References
*Lactobacillus reuteri*	Animal	Attenuation of antisocial behavior in male Shank3 KO and repetitive behavior in both male and female Shank3 mouse model	Tabouy et al., 2018
*Lactobacillus acidophilus*	Human	Decreased D-arabinitol/L-arabinitol which is responsible for carbohydrate and mineral malabsorption in ASD individuals	Kałużna-Czaplińska and Błaszczyk, 2012
Mixed culture of bifidobacteria and *Lactobacillis* strains	Animal	PPA-hamster model resulted in changes in gut microbiota, amelioration of glutamate excitotoxicity and decreased excitatory neurotransmitters	El-Ansary et al., 2018
General probiotics	Human	Improvement in school performance and food choices	Grossi et al., 2016
*Lactobacillus plantarum* WCFS1	Human	Improved anti-social behavior, anxiety and communication problems in children with ASDs	Parracho et al., 2010
VSK#3: Bifidobacteria (*B. longum, B. infantis*), lactobacilli (*L. acidophilus, L. delbrueckii* subsp. *L. bulgaris* and *L. plantarum*) and *Streptococcus salivaris* subsp. *thermophilus*	Human	Gastrointestinal disturbances and neurobehavioral symptoms were reduced in a 12-year boy with ASD	Pärtty et al., 2015
Vivomixx probiotics: *Streptococcus thermophilus*, *Bifidobacterium breve*, *Bifidobacterium longum,* *Bifidobacterium infantis*, *Lactobacillus acidophilus*, *Lactobacillus plantarum*, *Lactobacillus paracasei*, *Lactobacillus delbrueckii* subsp. *bulgaricus*	Human	Electroencephalogram (EEG) changes in brain activity (improved balance between excitatory and inhibitory neurons) in children with ASDs and changes in levels of serum lipopolysaccharide, leptin, TNF, IL-6, PAI-1, fecal calprotectin	Billeci et al., 2022
3 Strains: *Lactobacillus acidophilus,* *Lactobacillus rhamnosus,* *Bifidobacteria longum*	Human	Improvement was observed in the severity of ASD by significant improvements language, speech, communication, sociability, sensory/cognition, health/physical/behavioral. Also, improvements in GI disturbances	Sichel et al., 2013
*Bifidobacterium infantis* in combination with a prebiotic Bovine Colostrum Product (BCP)	Human	Reduction in frequency of certain GI symptoms. Reduced occurrence of abnormal behaviors. Reduction in IL-13 and TNFα	Sanctuary et al., 2019
3 Strains: *Lactobacillus acidophilus*, *Lactobacillus rhamnosus* and *Bifidobacterium*	Human	Improved ASD-related aberrant behaviors and GI symptoms in children with ASD who received probiotics	Shaaban et al., 2018
2 Strains: *Bifidibacteria and Lactobacilli*		Improved ASD-related behaviors with alleviation of glutamate excitotoxicity by reducing glutamate	El-Ansary et al., 2018
*Bacteriocides fragilis*	Human	Improved social behavior and cognition deficits in children with FXS	Lin et al., 2020
Probiotic and oxytocin combination therapy	Human	Improved ASD core socio-behavioral phenotypes and reduced gut microbiome dysbiosis	Kong et al., 2021

Probiotics have shown potential for the treatment of ASDs and are well tolerated with no severe adverse side effects. In clinical trials, probiotics showed potential in treating ASD-like behavioral phenotypes and GI symptoms as well as sensory profiles in children with ASDs (Santocchi et al., 2020; Feng et al., 2021). However, there are considerable differences in microbial abundance, i.e., diversity and richness measurements, which may be due to differences in subject age, diet, race, and/or ethnicity; sampling methods; and analytical differences (Martínez-González and Andreo-Martínez, 2019).

Probiotics contains beneficial microorganisms that improve gut health, therefore restoring the gut microbiome should improve gut health. In a 2016 study, children with ASD exhibited a higher proportion of *Candida albicans* in their stool samples (Kantarcioglu et al., 2016) although Adams et al. (2011) found no notable difference in yeast content in children with ASDs (Adams et al., 2011). An increase in D-arabinitol (DA) in the urine of children with ASDs was used as a marker of therapeutic effectiveness of probiotic treatments. The authors found a significant decrease in DA levels in urine after supplementing with probiotics (*Lactobacillus acidophilus*) (Kałużna-Czaplińska and Błaszczyk, 2012). Children with ASDs and lower levels of *Lactobacillus* experience increased lactulose absorption resulting in aberrant bowel movements and constipation (Iovene et al., 2017). Conversely, other studies have shown higher levels of *Lactobacilli* in subjects with ASDs (Strati et al., 2017). These inconsistencies highlight the importance of further research to define altered gut microbiota composition in ASDs to better understand how supplementation may impact disease phenotypes.

In 2010 study, 22 children aged 3–16 years were given the probiotic, *Lactobacillus plantarum* WCFS1, for 12 weeks and showed improved GI pathologies and significant amelioration of ASD behaviors. This study also reported an increase in *Lactobacillus* and *Enterococci* with a decrease in *Clostridium coccoides* in children with ASD compared to the placebo group (Parracho et al., 2010).

A 12-year-old boy with ASD and severe cognitive deficits was supplemented with VSL#3, which is recommended as a general probiotic, containing a mixed culture of 10 probiotics for 4 weeks. This treatment unexpectedly improved aberrant behavior associated with ASD. However, the authors recommend pursuing further research to truly uncover the efficacy of VSL#3 to treat ASD-related complexities through large scale and controlled studies (Grossi et al., 2016). In 2013, children with ASDs were provided with a mixture of 5 probiotic strains and treatment showed significant improvements in all domains of the autism treatment evaluation checklist (ATEC) along with amelioration of GI disturbances (Sichel et al., 2013). In a 2022 study, scientists explored the effects of vivomixx using electroencephalography (EEG). EEG detects abnormal brain activity related to seizures and sleep. Children with ASDs who received vivomixx showed improvement in neuronal excitatory/inhibitory balance (Billeci et al., 2022). Children 5–9 years old and diagnosed with an ASD received probiotics containing 3 strains i.e., *Lactobacillus acidophilus, Lactobacillus rhamnosus* and *Bifidobacterium* once daily for 3 months. Consistent with the previous studies, they showed improvement in ASD-like behaviors and GI symptoms (Shaaban et al., 2018). Interestingly, a combined oxytocin-probiotic therapy was given to individuals (3–20 years) with ASDs. Subjects received *Lactobacillus plantarum* PS128 probiotic for 28 weeks, while on the 16th week subjects started receiving oxytocin along with the probiotic strain. Results showed significant improvements in socio-behavioral traits along with amelioration in GI disturbances (Kong et al., 2021). Both ketogenic and FODMAP diets altered gut microbiota in a 17-year-old girl with ASD and epilepsy, but the KD was poorly tolerated while the low fermentable oligosaccharides, disaccharides, monosaccharides, and polyols (FODMAP) diet was well-tolerated and resulted in significant improvement in neurological, intestinal, and metabolic symptoms (Bertuccioli et al., 2022).

In preclinical studies, animal models of ASD indicate that probiotics have beneficial effects on mood swings and GI problems (Mangiola et al., 2016). A rat model with valproic acid-induced ASD treated with probiotic mixture (*Lactobacillus* spp*., Bifidobacterium* spp.) showed reduced antisocial behaviors and alteration in the gut microbial population (Mintál et al., 2022). Probiotic therapies have also been used in maternal immune activation (MIA) mouse models of ASD. Pregnant mice are injected with 20 mg/kg poly (I:C) and the offspring develop core ASD phenotypes including deficits in communication and social behaviors. Offspring are then treated with a single strain probiotic treatment, *Bacteroides fragilis*, which ameliorates GI problems and improves communicative, repetitive anxiety-like, socialism, sensory profile, and stereotyped behaviors (Hsiao et al., 2013). *L. reuteri* has also been shown to attenuate anti-socialism in male *Shank3*^KO^** mice while repetitive behavior was ameliorated in both male and female mice (Tabouy et al., 2018). Oral supplementation of probiotics (*B. bifidum, B. infantis*, and *Lactobacillus helveticus*) in C57BL/6J pregnant mice every 24 h for up to 21 days along with prebiotics (fructooligosaccharides and maltodextrin) improved ASD like characteristics in offspring (Abdellatif et al., 2020). Autistic behaviors were induced in juvenile hamsters with clindamycin and propionic acid (PPA) followed by supplementation with *Bifidobacteria* and *Lactobacillus* strains resulting in mitigation of glutamate excitotoxicity (El-Ansary et al., 2018). FXS is the most common genetic cause of ASDs; however, the consideration and application of microbiomal supplementation as a therapeutic approach is relatively new. One study showed *Bacteriocides fragilis* supplementation can improve ASD-like abnormal behaviors including social and cognitive deficits in *Fmr1*^KO^** mice (Lin et al., 2020).

## Behavioral interventions

Behavioral interventions have been utilized to combat negative attitudes toward food intake in ASDs. Several behavioral techniques have been used including applied behavioral analysis (ABA). ABA is a strategy to understand and alter the behaviors of young children with ASDs. ABA itself is not a therapy but a combination of different strategies and techniques to help people learn new skills and behaviors. The ABA approach has been shown to reduce food refusal and food selectivity while increasing interest in novel food, ultimately promoting consumption of diverse nutrients in children with ASDs. In addition, social story interventions have gained attention among behavioral scientists to improve social skills in people with ASD. Social story intervention is a technique where stories are used to increase engagement in socially appropriate mealtime attitudes in children with ASDs (Ozdemir, 2010). Others have applied social story techniques to children with Asperger syndrome, a type of ASD, and saw improvements in adequate dietary intake and overall nutritional status (Bledsoe et al., 2003). Sensory-based therapies have also been useful in developing appropriate mealtime behaviors in a closely monitored environment (Geraghty et al., 2010), although those therapies have not been applied to people with ASDs. Moving forward, it will be important to utilize complementary approaches including ABA and diet to improve the lives of persons with ASDs.

## Future directions

Progress in ASD research, particularly in FXS, has provided a better understanding of the core symptoms and identification of potential treatment options. However, differences in underlying mechanisms leading to convergent and divergent phenotypes between FXS and ASDs as well as lack of consistent results in both preclinical and clinical studies are important issues. In addition, the major focus of research in neurodevelopmental disorders is the brain with minimal efforts aimed at the other organ systems or a whole-body approach. A plethora of nutritional interventions have been assessed in both animal and human models without conclusive results. Generally, small sample sizes, lack of appropriate controls, ambiguous results, and lack of technical or economic support limit preclinical, clinical, and translational ASD nutrition studies. Moreover, translation of nutritional interventions from animals to humans yields inconsistent results. Animal testing designs may not always be applicable for human trials due to genetic and epigenetic factors, subject variation and altered metabolism between species.

We propose the following future directions to address rigor and reproducibility in and promotion of nutrition research related to ASDs: (1) Publications must report details on rodent diet. Only 13% of publications reporting behavior results in *Fmr1*^*K*O^ mice identified the rodent diet in the Methods section of their papers (Ripp and Westmark, unpublished results). (2) Nutritional interventions should be tested in multiple rodent models of ASDs to assess the generalizability of the findings. There are a plethora of genetic mutations contributing to ASDs and it will be important to identify diet-induced effects as a function of specific autism mutations, behaviors and biomarkers to understand both complementary and divergent mechanisms. (3) The mechanism(s) underlying diet effects need to be determined, i.e., microbiota composition, gut permeability, immune system activation, epigenetics, mitochondrial function, etc. as a function of autism genetic mutations and diet. Of note, there is a growing body of knowledge regarding mitochondrial dysfunction in ASDs including FXS (Shen et al., 2019; Licznerski et al., 2020; Frye et al., 2021). And (4) it is necessary to assess sex-specific differences and long-term effects. A growing body of literature suggests that females require a “bigger genetic hit” than males before developing autism (Turner et al., 2019; Schenkman, 2020). There is minimal literature regarding long-term behavior outcomes as a function of infant diet. Ultimately, prospective evaluation of neurodevelopment as a function of ASD mutations and diet would further the field. In conclusion, consideration of the above factors will lead to a better understanding of effective nutritional interventions for the treatment of ASDs including FXS. This avenue of research is significant considering that choice of infant and early childhood feeding could reduce the development and/or severity of ASDs.

## Author contributions

All authors contributed to the writing and editing of the manuscript.
